# Enzymatic digestion of hair increases extraction yields of cortisol: a novel two-dimensional liquid chromatography-tandem mass spectrometry method for hair cortisol analysis

**DOI:** 10.1007/s00216-025-05878-0

**Published:** 2025-04-16

**Authors:** Dewi van Harskamp, Mariëtte T. Ackermans, Wjera V. Wickenhagen, Annemieke C. Heijboer, Johannes B. van Goudoever

**Affiliations:** 1 Department of Laboratory Medicine, Core Facility Metabolomics, Laboratory Genetic Metabolic Disease, Amsterdam UMC, University of Amsterdam, Meibergdreef 9, 1105 AZ Amsterdam, Netherlands; 2 Emma Center for Personalized Medicine, Amsterdam UMC, Amsterdam, Netherlands; 3 Amsterdam Gastroenterology, Endocrinology & Metabolism, Amsterdam UMC, Amsterdam, Netherlands; 4 Ouderkerk aan de Amstel, Netherlands; 5 Department of Laboratory Medicine, Endocrine Laboratory, Vrije Universiteit Amsterdam, Amsterdam UMC, University of Amsterdam, Amsterdam, Netherlands; 6 Amsterdam Reproduction & Development Research Institute, Amsterdam UMC, Amsterdam, Netherlands; 7 Department of Pediatrics, Emma Children’s Hospital, Vrije Universiteit Amsterdam, Amsterdam UMC, University of Amsterdam, Amsterdam, Netherlands

**Keywords:** Chronic stress, Hair cortisol concentration, Liquid chromatography, Mass spectrometry, Proteinase K for digestion of hair, Retrospective

## Abstract

Hair cortisol concentration is a retrospective, long-term measure of cortisol secretion, often used as a biomarker for chronic stress or suspected cyclic Cushing’s syndrome. Various methods for hair cortisol extraction, typically involving intact, minced, or milled hair incubated with methanol or extraction buffers, may not achieve complete extraction. Incomplete extraction can bias results, particularly when comparing hair samples of differing thickness and texture. Additionally, sample-to-sample variation might be caused by inconsistent cutting or grinding of the hair by different technicians. We aimed to achieve complete cortisol extraction through the enzymatic digestion of hair. After digestion and sample clean-up, we analyzed the final extracts using two-dimensional liquid chromatography-mass spectrometry. Complete digestion was observed with an overnight enzymatic reaction. This method demonstrated high reproducibility, with an intra-day precision of 3.6%, and inter-day precision of 6.5% in a homogenized pool. Moreover, duplicate CV% of intact hairlocks ranged from 0.1% to 8.9%. Our protocol does not influence the cortisol concentration. It is less labor-intensive and more consistent than mincing or milling hair. Our extraction yield was higher compared to the commonly used methanol extraction method. Samples extracted with methanol showed, on average, 19% lower cortisol concentrations than those measured in digested aliquots. We have developed and validated a new, superior method for extracting cortisol from hair utilizing enzymatic digestion. This strategy provides a more thorough extraction, greater consistency, and requires less labor than traditional methods.

**Graphical Abstract:**

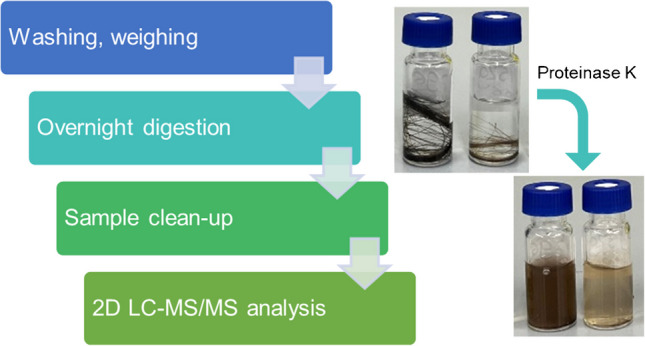

**Supplementary Information:**

The online version contains supplementary material available at 10.1007/s00216-025-05878-0.

## Introduction

Chronic stress has a long-lasting impact on health [1–4], and even maternal stress during pregnancy can have profound long-term effects on the child’s development and disease risk [5, 6]. Researchers are delving into the role of stress in the development of diseases, while also searching for ways to alleviate stress and its consequences. Given that questionnaires and self-reporting tools used to quantify stress are limited by personal perception and recall bias, there exists the need for an objective biomarker to ascertain the degree of stress individuals undergo.

In response to stress, the adrenal cortex produces glucocorticoids, including cortisol. An acute increase in cortisol levels triggers the fight-or-flight response, a beneficial survival mechanism. However, high levels maintained over prolonged periods can alter metabolism homeostasis. This, over time, can lead to the development of conditions such as obesity, metabolic syndrome, and cardiovascular disease [2]. During chronic stress, cortisol levels do not return to a normal low level but remain elevated for an extended period. This makes cortisol a potential stress biomarker [3]. Yet, it is challenging to measure cortisol levels due to its circadian release pattern and the influence of acute events like injury, exercise, or food intake which strongly affect cortisol levels. Considering the effects of the circadian rhythm and acute situational impacts on cortisol blood or saliva levels, these measurements are not suitable as a reliable stress marker. Short-term cortisol secretion can be assessed through 24-h urine cortisol levels analysis, but this method does not serve as a marker for chronic stress.

Hair cortisol has been identified as a viable biomarker for chronic stress [7], with the hair matrix serving as a tool to provide a long-term retrospective overview of an individual’s past cortisol levels [8]. This is because cortisol is continuously incorporated into growing hair. Hair analysis offers some unique features compared to traditional sample matrices. As summarized by Stalder et al. [7], these include the ability to measure long-term cortisol secretion, the opportunity to analyze cortisol levels retrospectively, non-invasive sampling, and simple storage at room temperature. Over the past 5 years, at least 1065 manuscripts have been published discussing hair cortisol measurements (PubMed search terms: hair cortisol; period 2020–2024; consulted December 2024).

Long hair segments have been used to determine long-term cortisol secretion patterns [9]. However, it is argued that hair cortisol analysis is only reliable in the first 6 cm of the proximal segment [10], and some limit this to the first proximal 3 cm segment [11–13]. The inconsistent results observed in distal segments are possibly due to hair washing or environmental factors such as sunlight exposure [14]. A recent publication reported a negative decline within the first 3 cm measured from the scalp [15]. This is an important factor to consider while using this as a biomarker for pathological conditions like Cushing’s syndrome. In studies on chronic stress, the cortisol level of the first 3 cm is often reported, and results are compared across groups, thus averaging out this decline. By measuring 3 cm instead of shorter hair segments, the results are less influenced by temporary increases that are not linked to chronic stress.

While individuals display variations in hair growth rates, it is typically assumed that the average rate is 1 cm/month [16]. Consequently, it is theoretically possible to retrospectively measure cortisol secretion for the 3 months before sampling. The feasibility of chronological segmentation analysis has been demonstrated (e.g., [9]), yet it remains a topic of debate due to the above-mentioned issues. Furthermore, the accuracy of period attribution per segment is affected by variations in the growth rate [17].

Various methods have been documented for the extraction of cortisol from hair. Many protocols involve the pulverization or mincing of hair to enhance the surface area in contact with the extraction solvent. However, these processes tend to be labor-intensive and lack standardization; therefore, the final result often hinges on the technician’s skills and effort. Milling or grinding hair are alternative procedures for increasing the surface area, but they are considered suboptimal due to potential risks, such as partial sample loss, degradation, and increased possibility of sample carryover [17].

Our hypothesis suggests a risk of incomplete cortisol extraction with the prevailing procedures. Validation is hampered by the inability to accurately determine the extraction yield since the external addition of cortisol fails to mirror the amount genuinely extracted from the hair structure. Spiking a hair sample to represent endogenous hair cortisol accurately is unfeasible. Literature [18] provides evidence supporting our concerns about the incomplete extraction yield of cortisol from the hair structure. Biased outcomes may result from incomplete extraction, as the extraction recovery could depend on individual hair traits like thickness and texture, varying per hair type. Should the extraction of cortisol from the hair be complete, no potential difference in extraction yield among samples would occur, leading to more accurate, precise, and reproducible quantification.

We sought to achieve total extraction of cortisol from hair samples by dissolving the hair via enzymatic digestion. This method has prior applications in the quantitative analysis of drugs in the hair for therapeutic drug monitoring [19–21]. We refined the sample preparation procedure and the two-dimensional liquid chromatography-tandem mass spectrometry (2D LC–MS/MS) method and validated the analytical process. Employing the new procedure, we contrasted our technique with the methanol extraction of the hair (considered the current gold standard). We speculate that if we can accurately reliably measure cortisol—that is, unaffected by hair type or the technician preparing the samples—it could enhance hair cortisol analysis. This advancement could ultimately assist clinical researchers in objectively verifying their efforts to reduce stress or investigate its role in the development of diseases.

## Methods

**Fig. 1:**
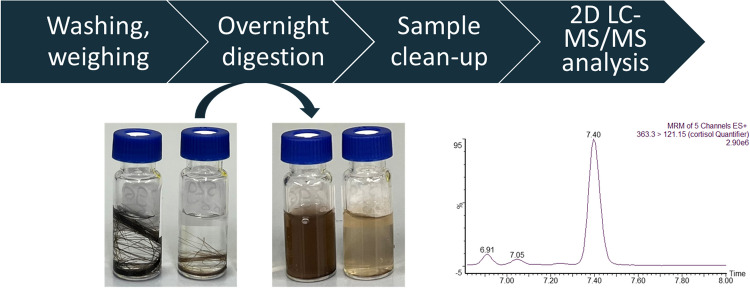
Schematic depiction of sample processing steps

To optimize extraction yield, hair was completely dissolved through enzymatic digestion. Once the samples were prepared, the final extracts were then analyzed using liquid chromatography-tandem mass spectrometry, as this is the current best practice for cortisol analysis. The workflow is depicted in Fig. 1. We used a two-dimensional liquid chromatography-tandem mass spectrometry (2D LC–MS/MS) method for quantifying cortisol in the digested hair samples. Experiments were then conducted to compare the extraction yield of this new approach with methanol extraction of minced hair. After optimization, the protocol was then validated.

### Reagents

Cortisol (Cerilliant, Round Rock, TX, USA), ^13^C_3_-cortisol (99% ^13^C), 1,4-dithioerythritol (DTE) (Sigma-Aldrich, Darmstadt, Germany), Tris base (Roche, Basel, Switzerland), potassium chloride, and proteinase K (Merck, Darmstadt, Germany), along with isopropanol (ULC/MS grade), tert-Butyl methyl ether (MTBE, HPLC grade), methanol (ULC/MS grade), and formic acid (ULC/MS grade) (Biosolve, Valkenswaard, The Netherlands), were all used in the experiments. Furthermore, ammonium acetate (HPLC grade, Honeywell Fluka, Charlotte, NC, USA) and sodium dodecyl sulfate (SDS, Fisher Scientific, Waltham, MA, USA) were also used. For each experiment, the digestion buffer was freshly prepared, containing 2 mg/mL of proteinase K, 6 mg/mL of DTE, and 20 mg/mL of SDS in a Tris buffer (0.1 M, pH 7.2).

### Sample collection

For the validation experiments, hair samples were acquired, following informed consent, from adult female volunteers (*n* = 26) and drop-outs of a clinical study (*n* = 43). In this study, healthy lactating females from the Netherlands, all aged over 18 years, were included. A lock of hair (consisting of 100–200 strands) was snipped with scissors from the posterior vortex as close to the scalp as possible. Each hair sample was carefully attached to a piece of paper with tape to indicate the scalp end. The samples were stored in envelopes at room temperature in a dark closet until analysis [8]. The set of samples included a variety of hair types with varying thickness, texture (ranging from straight and fine to curly and coarse), color (from blond, brown, black, red, to gray), and also some dyed hair samples. For analysis, an average of 10.6 mg of hair was prepared from each sample.

### Standard preparation

Stock solutions of cortisol and ^13^C_3_-cortisol (internal standard) were prepared in ethanol and stored at 4 °C. A calibration curve was created by adding dilutions of the cortisol stock to a fixed amount of the internal standard, to cover a relevant range (6–574 pg). Calibration standards were dried under a gentle stream of nitrogen and reconstituted in 60 µL of 20% methanol in water.

Hair results are initially computed as an absolute amount in picograms (pg), which is subsequently corrected for the precise weight used in the preparation and reported as picograms per milligram of hair (pg/mg hair). For easier calculation and correction of the initial results, the calibration curve covers a range from 6 to 574 pg. This represents the quantity of cortisol used in the preparation, without any correction for the hair weight.

### Control samples

A significant quantity (approximately 10 g) of hair was gathered, using only the first 3 cm measured from the scalp. It was then washed with isopropanol. After stirring it for 1 min, the washing fluid was poured off, and the hair was left to dry for at least 4 days at room temperature in a fume hood to make sure of complete evaporation. The hair was finely cut and mixed to ensure a homogeneous pool. Note that this cutting process was only to guarantee the uniformity of a large volume of hair used as a control pool for quality control purposes and deviates from the standard preparation protocol for hair samples, where intact locks are taken into preparation. The finely cut, well-mixed pool of hair was stored in room temperature conditions without light exposure. Aliquots were weighed separately on analysis days to serve as control samples.

### Sample preparation protocol

The first 3 cm of hair lock, measured from the scalp, was cut and transferred into a glass tube. Isopropanol (2 mL) was added to each sample, which was then vortexed for 1 min. After centrifugation for 1 min at 3000 × g, the fluid was decanted, and as much liquid as possible was removed from the tubes. The outer walls of the tubes were then covered with aluminum foil; this reduced exposure to light to prevent potential cortisol degradation while keeping the opening free to allow any leftover isopropanol to evaporate. The samples were placed at room temperature in a fume hood for 4 days.

The washed hair locks were accurately weighed into silanized vials using an analytical balance (ME204 T, Mettler Toledo, Netherlands). An internal standard (10 µL of 23.5 pg/µL ^13^C_3_-cortisol in ethanol = 235 pg absolute) and 1 mL of digestion buffer were added to each vial. The samples were then incubated at 40 °C for 16–40 h. After incubation, the digested hair samples were transferred into borosilicate tubes. A small amount of potassium chloride (about 0.15–0.2 g) was added and thoroughly mixed by vortexing. The samples were then incubated at 4 °C for 1 h.

To extract cortisol from the digest, 2 mL of MTBE was added. The samples were then vortexed for 1 min, incubated at − 20 °C for 5 min, and centrifuged for 5 min at 4 °C with 3000 × g. The top layer was transferred into a clean silanized vial, dried under a gentle nitrogen stream at 50 °C, and reconstituted in 60 µL of 20% methanol in water. This was then transferred into a total recovery vial and analyzed on 2D LC–MS/MS.

#### 2D LC–MS/MS

A Waters LC–MS system (Waters Acquity UPLC equipped with a binary solvent manager, a quaternary solvent manager, a sample manager, a column heater, and a Xevo TQ-S, Waters, Milford, USA) was utilized for all analyses. This system was operated in 2D LC mode, featuring a Protein BEH C4 column (1.7 µm, 2.1 × 50 mm, Waters) as the first column, and an Acquity HSS T3 column (1.8 µm, 2.1 × 50 mm, Waters) as the second column. After injecting a 10 µL sample, separation was achieved by a gradient with two mobile phases: mobile phase A, composed of 2 mM ammonium acetate and 0.1% formic acid in water, and mobile phase B, composed of 2 mM ammonium acetate and 0.1% formic acid in methanol. The LC gradients can be found in the supplementary material. Between 4 and 5 min, the effluent of the first column was directed to the second column, with cortisol eluting after 7.4 min. The total duration per process was 10.5 min.

Cortisol and ^13^C_3_-cortisol were recorded by the Xevo TQ-S, which operated in MRM mode (transitions were 363 > 121 and 366 > 124, respectively). Data acquisition was carried out using MassLynx V4.2 SCN1001. Additional details on the instrument settings can be found in the supplementary material.

### Method validation

#### Precision

The intra-day precision was determined by analyzing a homogenized control pool that was prepared repeatedly on a single day (*n* = 16). The inter-day precision was assessed by analyzing this same pool, which was prepared repeatedly on different days (5 days, *n* per day ≥ 2). From these data, we calculated the coefficient of variation (CV%).

Additionally, hair locks from volunteers with various hair types were gathered. After washing, the hair was divided into two aliquots for duplicate analysis. Based on these results, the CV% was calculated.

### Recovery, matrix effect

We cannot determine the true extraction yield, as there is no means to verify the exact endogenous concentration in hair samples. Current techniques may be biased, and spiking does not accurately represent endogenous hair cortisol. By completely digesting the hair and eliminating any solid structure, we are confident that we have maximized the extraction yield, if not achieved 100%.

The recovery of sample preparation was assessed by adding IS before and after preparation of a hair sample. The matrix effect was evaluated through the analysis of a prepared hair sample, spiked with IS after sample preparation, and the analysis of the same amount of IS added to the solvent.

Additionally, a spiking experiment was performed, in which several locks of hair were divided into aliquots. For each lock of hair, one unspiked aliquot was analyzed to determine the concentration in the respective hair sample, while two or three aliquots were spiked with cortisol before digestion.

The equations required for the calculation of these parameters are presented as supplementary material.

#### Linearity

The standard curve ranged from 6 to 550 pg, an *R*^2^ of > 0.99 is acceptable. Sample weights ranging from 5.8 to 38.6 mg of hair from the pool were analyzed to determine whether the sample size affected the outcome. The measurement results were then plotted against the theoretical outcomes based on the starting material’s weight. A slope between 0.9 and 1.1 was deemed acceptable, along with an intercept of less than 25% of the lowest observed result.

To determine the accuracy of the final extract’s dilution, a sample with a high cortisol level was diluted in a series: 1:1, 1:3, 1:7, 1:15, and 1:31 (sample:20% methanol in water).

#### Limit of quantification

The limit of quantification was determined from a tenfold analysis of both a sample and a standard with a low cortisol concentration. A CV% of less than 20% is acceptable for the lower limit of quantification.

#### Stability

The stability of the samples was assessed over a relevant time frame after being prepared. Multiple samples were prepared and pooled to ensure ample material for repeated injections on days 0, 2, 7, 9, and 14 post-preparation. In between runs, these samples were stored at 4 °C. The CV% of these results should be less than 10%, consistent with the acceptable limit for inter-day precision.

#### Carryover

Carryover was assessed using the statistical module specifically designed for carryover on the EP Evaluator software (Data Innovations LLC, Colchester, USA, Build 12.3.0.2), based on the Clinical & Laboratory Standards Institute (CLSI) EP10 guideline. Two samples, one with a high and one with a low concentration of cortisol, were repeatedly analyzed in a prescribed order according to the module’s instructions.

### Comparison to methanol extraction

To compare the results of digestion to those obtained through the commonly utilized methanol extraction technique, eight locks of hair were divided into two aliquots after the isopropanol washing step. One aliquot from each lock underwent digestion following the procedure detailed in the “Sample preparation protocol” section, while the other was treated using the following method:

The hair was finely cut with scissors and then incubated in 1 ml of methanol for 16 h at 52 °C. Afterward, the samples were cooled and centrifuged for 5 min at 3000 × g. The supernatant was decanted and dried under a gentle stream of nitrogen. This methanol extraction was based on the publication by Noppe et al. [22]. The remaining steps were based on a novel sample preparation procedure to ensure result comparability: The residue was dissolved in 0.5 mL water, onto which 2 mL of MTBE was added. The samples were then vortexed for 1 min and centrifuged for 5 min at 4 °C 3000 × g. The top layer was transferred into a clean silanized vial, dried under a gentle stream of nitrogen at 50 °C, and reconstituted in 60 µL of 20% methanol in water. This was then transferred into a total recovery vial and analyzed with 2D LC–MS/MS.

### Data analysis

The peak area was integrated using TargetLynx software version V4.2 SCN1001 (Waters, USA). Further calculations were carried out on Excel 2016 (Microsoft Corporation, USA). The slope and intercept of the standard curves were derived from linear regression analysis. For the comparison of methods and evaluation of volunteers’ results, MedCalc® Statistical Software version 22.016 (MedCalc Software Ltd, Ostend, Belgium; https://www.medcalc.org; 2023) was used.

## Results

Complete digestion of hair through an overnight enzymatic reaction was observed under the protocol conditions we proposed. The hair structure was completely dissolved by proteinase K digestion, resulting in a colored solution devoid of any solid structures or particles visible to the naked eye (Fig. 1). This strategy proved highly reproducible, and our protocol was successful across all analyzed hair types.

### Enzymatic digestion

The enzymatic digestion protocol was based on a protocol reported in the literature [23]. We found the optimal conditions by varying the composition of the digestion buffer. Notably, SDS and 1,4-dithiothreitol (DTT) interfered with the subsequent 2D LC–MS/MS protocol. DTT was successfully replaced with DTE, achieving efficient digestion and cleaner chromatography. However, we did not find a suitable alternative for SDS that did not affect digestion. To lower the SDS concentration in the final extract, we added a precipitation step with potassium chloride followed by incubation at a low temperature to the sample preparation procedure. This was found to reduce the matrix effect and ion suppression.

In addition, the type of tubes used for holding the hair and digestion buffer influenced the completeness of the digestion process. For instance, “Protein Lobind” Eppendorf tubes or borosilicate tubes with a round bottom were found to be unsuitable. Conversely, the use of silanized GC vials featuring a flat bottom and a screw cap proved compatible with the digestion procedure.

Digestion was always complete after 16 h of incubation. However, the stability of cortisol in the digest was confirmed up to at least 40 h. This enables more flexible planning of experiments.

### Method validation

#### Precision

The intra-day precision (*n* = 16) was 3.6 CV%, and the inter-day precision (5 days, *n* ≥ 2) was 6.5 CV%. These values were determined from repeated sample preparations of a single, homogenized pool. The average observed concentration in this pool was 7.98 pg/mg. 

**Fig. 2:**
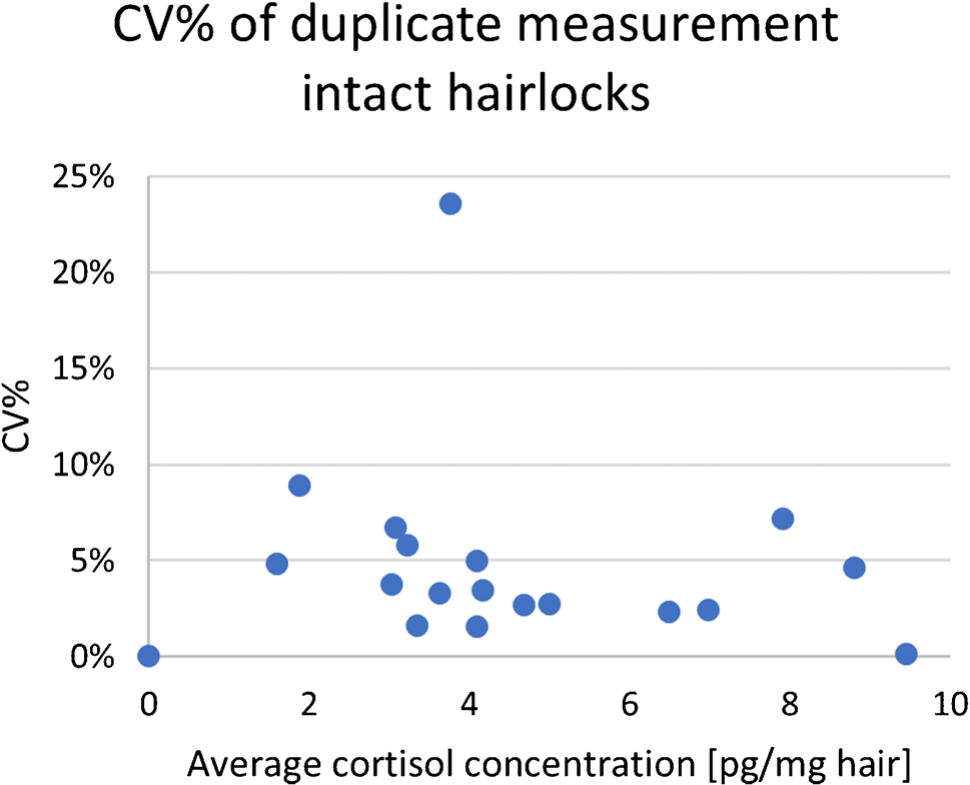
Variation coefficient (CV, expressed as %) of duplicate measurement of hair locks from different hair types, results are summarized in Table 1

Additionally, the CV% from duplicate analyses of intact hair locks from 18 volunteers with varied hair types was shown in Table 1 and Fig. 2. These hair types were divided into two aliquots for repeated analysis after washing. The CV% of the duplicate analyses ranged from 0.1% to 8.9%, with one outlier at 23.6% in the 3–5 pg/mg hair result range. The results included in the table take this outlier into account.

**Table 1:** CV% of duplicate measurement of hair locks from different hair types

Result range	Average result	Number of samples within result range	CV% of duplicate measurement
1–3 pg/mg hair	1.7 pg/mg hair	2	7.5%
3–5 pg/mg hair	3.7 pg/mg hair	10	8.4%
5–10 pg/mg hair	7.4 pg/mg hair	6	4.1%

#### Recovery, matrix effect

The recovery of the sample preparation was 58% using this novel method. This indicates that 42% of the cortisol is lost during the preparation steps; however, this loss is adjusted by adding the stable isotope-labeled internal standard (^13^C_3_-cortisol) at the start of the process. This labeled (internal standard) and unlabeled (endogenous) cortisol experience identical losses during the preparation, so their ratio remains consistent throughout the procedure and is then used for quantification.

The matrix effect was 65%. The total of the matrix effect and recovery from sample preparation was also calculated from the results and was found to average 36%, ranging between 30–40% across various measurement days. These effects are accounted for by using the stable isotope-labeled internal standard as well.

During the spike experiment, unlabeled cortisol is added to a hair lock. A portion of this hair lock is analyzed without the spike of unlabeled cortisol to determine the endogenous cortisol concentration, which is used to adjust the result of the spike experiment. The recovery of the spike is calculated by dividing the observed spike, derived from the corrected sample result accounting for the endogenous amount of cortisol present, by the theoretical amount of cortisol added to the sample. This value should be approximately 100%, thereby assessing the accuracy of the method. The results of the spike experiment appear in Table 2.

**Table 2:** The spike experiment in various hair samples to determine the recovery of hair samples spiked with cortisol prior to digestion

Hair sample weight range	Concentration range in unspiked hair samples	Spike amount	Observed spike amount	Spike recovery
7.8–12.2 mg	4.66–6.59 pg/mg	14 pg	14–20 pg	97–137%
6.4–17.2 mg	0.90–8.14 pg/mg	57 pg	58–65 pg	101–115%
7.2–16.6 mg	0.90–8.14 pg/mg	144 pg	142–159 pg	99–111%

#### Linearity

The standard curve ranged from 6 to 550 pg, achieving an *R*^2^ of > 0.999 in each repetition. It expressed the absolute amount of cortisol used in the preparation. For hair samples, we need to calculate the absolute amount present, which is then corrected for the exact weight of the aliquot taken into preparation. The slope varied by 6% across 5 days. All hair analysis results fell within the curve’s range.

**Fig. 3:**
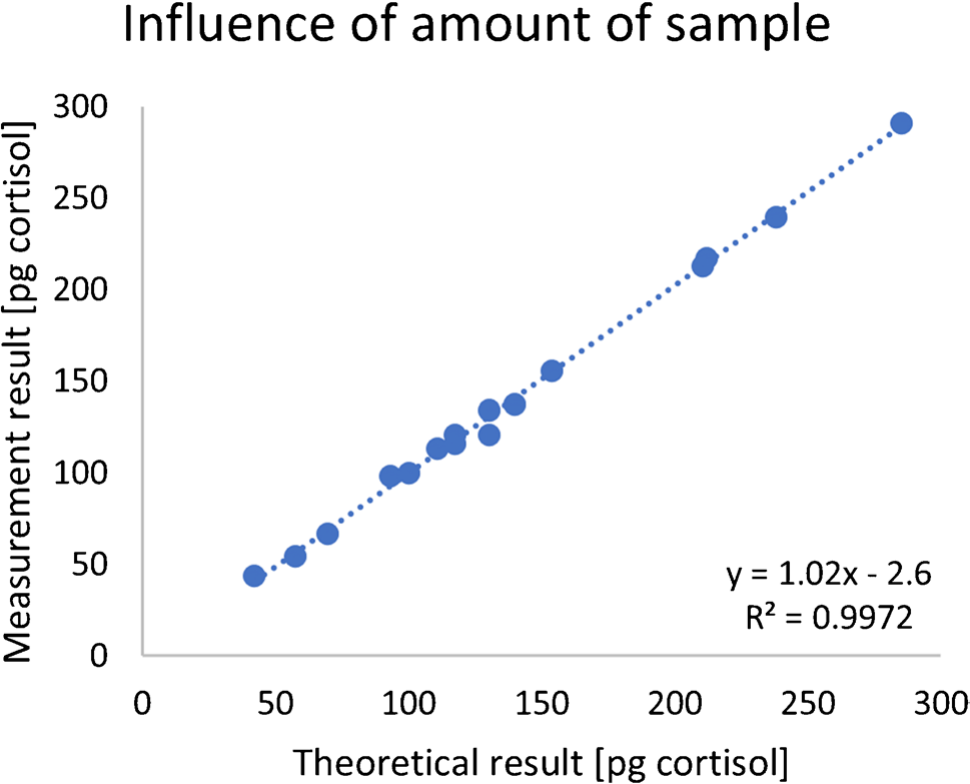
Repeated analysis of a homogenized hair pool with a wide range of starting material (5.8–38.6 mg hair). The measurement result is plotted against the theoretical result as calculated from the concentration of cortisol in hair multiplied by the weight of the starting material

The results from the repeated analysis of the homogenized control pool, with a wide range of starting material (ranging from 5.8–38.6 mg hair, *n* = 16), are depicted in Fig. 3. The measurement results were plotted against the theoretical result (average observed concentration of cortisol in the pool, multiplied by the weight of the starting material). A slope of 1.02 was observed, while the intercept came out to be 2.6, i.e., under 25% of the lowest observed result (42 pg cortisol). After correcting for the amount of hair prepared, the average result was 7.53 pg/mg hair, with a CV of 3.6% (*n* = 16).

A dilution series was analyzed, with the final extract diluted by solvent—ranging from undiluted to 32 times diluted. The analysis showed a precision of 1.5 CV% in the concentration results and a highly linear response (*R*^2^ > 0.999) across this dilution range.

#### Limit of quantification

A sample, expected to yield a low concentration, was prepared as a reference for the lower limit of quantification (LLOQ) determination. This sample’s concentration was 42.5 pg, and its precision (CV%) was 1.1% (*n* = 10). In the analysis of samples from other volunteers, the smallest observed value was 9.2 pg. Due to an inadequate amount of sample material, a tenfold repeat analysis could not be performed. Instead, tenfold injections of low standards were employed to compute the LLOQ. The precision (CV%) of a standard concentration of 2.7 pg cortisol was 4.0%, while for the lowest standard point of the calibration curve (5.8 pg cortisol), the precision was 2.2% (both *n* = 10). These precision values fell comfortably below the defined LLOQ limit of < 20%. As a result, the LLOQ was defined as the lowest standard point of the calibration curve, suggesting that the actual LLOQ is even lower.

#### Stability

The stability of the samples after preparation was established using four pooled samples, measured on days 0, 2, 7, 9, and 14 following preparation. The calculated CV% fell within the predefined limit of less than 10% for all pools (Table 3). In this table, also the CV%, as calculated from the raw data (i.e., [peak area cortisol]/[peak area internal standard]), is represented, showing a lower CV% for the uncorrected results.

**Table 3:** Stability of samples up to 14 days after preparation. CV% as calculated from pg observed is calculated from the data corrected by the calibration curve prepared on the day of analysis. CV% as calculated from raw data is not corrected with a calibration curve, but calculated directly from the [peak area cortisol]/[peak area internal standard] results

Samples	Average pg cortisol observed	CV% as calculated from pg observed	CV% as calculated from raw data
Pool A	174	5.0%	2.0%
Pool B	131	4.8%	1.7%
Pool C	156	6.7%	1.4%
Pool D	119	8.6%	3.1%

#### Carry over

No carryover effect was detected. The amount of cortisol [pg absolute] observed in the high sample was 487 pg, and in the low sample, it was 63 pg. The average result of a low value directly after another low was 63.0 pg, while the average result of a low value directly after a high was 63.5 pg. The error limit was determined as three times the SD of the low results, which was found to be 0.86. Since the difference between a low after low and a low after high (0.55) did not surpass this error limit, the analysis successfully passed the carryover test according to the CLSI EP10 guideline.

### Comparison to methanol extraction

The results of the method comparison between enzymatic digestion and methanol extraction can be seen in Table 4. On average, less cortisol (81% [range 66–89%]) could be measured in the hair samples extracted with methanol, compared to the aliquots digested enzymatically.

**Table 4:** Cortisol concentration results obtained for hair samples divided into two aliquots, prepared either with methanol extraction of the hair, or with the digestion protocol Standard deviation is provided in brackets

Sample	Methanol extraction [pg/mg hair]	Enzymatic digestion [pg/mg hair]	Ratio (%) methanol/enzymatic digestion
1	2.40	3.36	71%
2	4.15	5.06	82%
3	4.27	5.03	85%
4	4.40	4.94	89%
5	4.73	5.86	81%
6	4.76	5.79	82%
7	12.71	19.13	66%
8	44.31	50.45	88%
Mean (SD)			81% (8%)

**Fig. 4:**
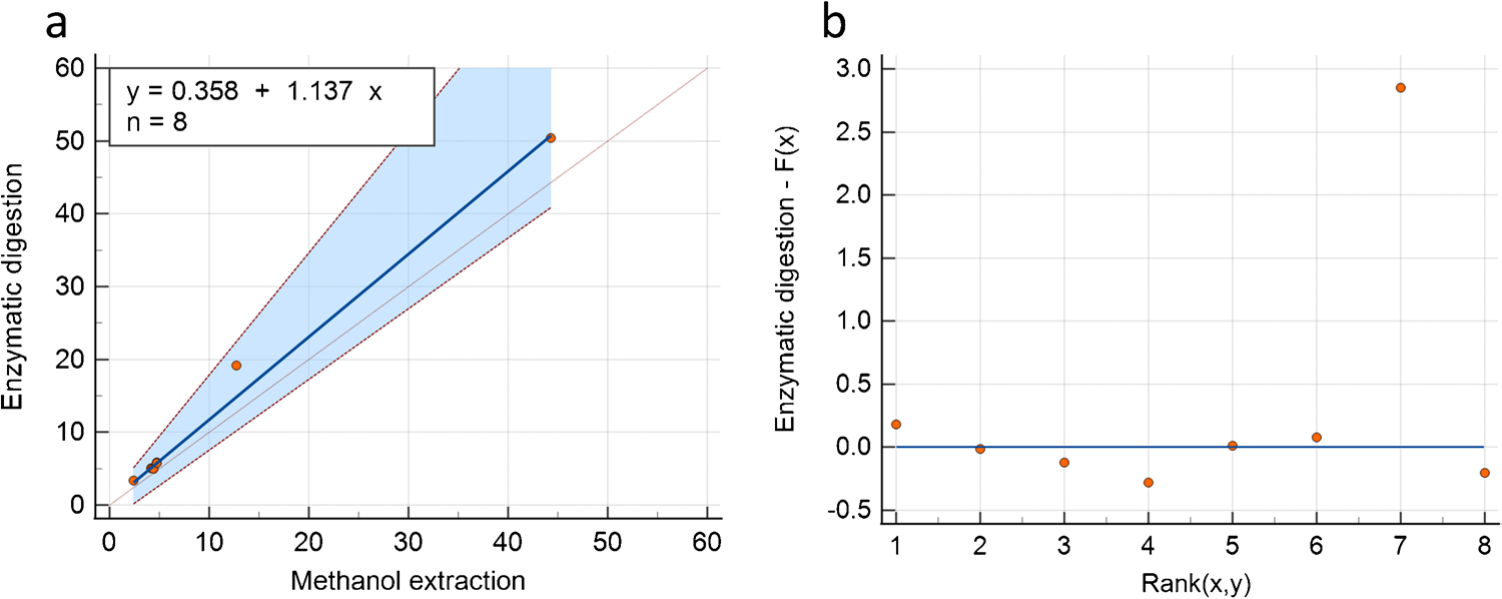
**a** Correlation of the results according to Passing Bablok regression. Comparison of results obtained with the methanol extraction of hair (*x*-axis) and the enzymatic digestion (*y*-axis) of the hair samples divided into two aliquots. The dotted line is the identity line, the continuous line the Passing and Bablok regression line. **b** The residual plot of the Passing and Bablok method comparison.

A Passing and Bablok analysis was performed using MedCalc software, for statistical analysis of the correlation of both methods, the resulting graph is depicted in Fig. 4. The following equation was found: *y* = 1.14*x* − 0.36 (95% confidence intervals; slope 0.9714 to 1.6780, intercept − 2.17 to 1.10) with no significant deviation from linearity (*p* = 0.63).

### Preliminary results of volunteers

**Fig. 5:**
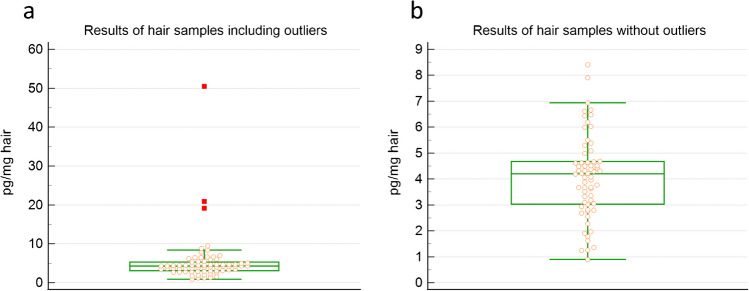
Distribution of the results of the hair cortisol analysis of samples from female volunteers (*n* = 70), including (**a**) and excluding (**b**) outliers (*n* = 5)

Hair samples were obtained from 69 female volunteers. The results for 64 samples ranged from 0.90 to 8.4 pg/mg cortisol in hair, with a median concentration of 4.20 pg/mg (25–75 percentile: 3.03 to 4.68) (Fig. 5).

Five results were identified as probable outliers by the Tukey method for outlier detection using MedCalc software (outside value: 8.76, 9.47 pg/mg and far-out values: 19.13, 20.87, 50.45 pg/mg). The highest value was obtained from a hair sample of a woman who confirmed experiencing a period of high stress. The statistical analysis of the results is represented in Table 5.

**Table 5:** Cortisol concentration results obtained for hair samples obtained from female volunteers, without the identified outliers

	*N*	Minimum	Maximum	Mean	Median	SD	RSD	25–75 P
pg_mg without outliers	64	0.900	8.400	4.086	4.200	1.6033	0.3924	3.030 to 4.675

## Discussion

We discuss the development and validation of a novel strategy designed to maximize cortisol extraction yield from hair. We show that our method is dissolving hair samples of varying texture and thickness, to very efficiently measure cortisol levels in a precise and repeatable manner. We propose that cortisol extraction from the hair matrix is complete upon total digestion. Incomplete extraction could lead to biased results and variability across hair types, while complete extraction will yield more accurate, precise, and reproducible quantification. Moreover, our technique is less time-consuming and more consistent compared to previously described protocols that utilize cutting or mincing practices, such as those detailed in [17, 22, 24, 25]. As a result, sample throughput and precision are improved. With former protocols, variations between samples could be caused either by inconsistent manual hair cutting by different technicians or by varying extraction yields due to differences in hair structure when intact, minced, or milled hair is incubated with methanol or extraction buffer.

Typically, a hair analysis procedure consists of washing off external contaminants, possibly pulverizing or mincing the sample, extracting cortisol from the processed hair, possibly cleaning up the sample, and then analyzing it using either LC–MS or ELISA. The latter is subject to potential cross-reactivity effects due to non-specific binding and cross-reactions of antibodies with similar steroid hormones [26]. Furthermore, it was developed for a different matrix (usually, kits developed for saliva analysis are used). We developed a new LC–MS/MS method and combined it with our new approach to extract cortisol from hair. We reduced the number of processing steps, as no separate mincing or pulverization is needed before the overnight digestion with proteinase K. After confirming the efficiency of the hair digestion using proteinase K, we needed to validate this approach in the analysis of cortisol. We found that the results for a cortisol standard curve were unaffected by the incubation protocol, in both the presence and absence of hair. Hair digestion is a process that is visible to the eye, as the structure of the hair dissolves over time, resulting in a colored solution, as can be seen in Fig. 1. After an incubation period of a few (1-4) hours, some solid hair residues were still present in the digests of all hair types. As additional sample preparation steps are required after digestion, we chose an incubation time of 16 h to allow the subsequent steps to be performed sequentially without interruption. This protocol is more efficient within the constraints of an 8-h working day. After 16 h (overnight) digestion, no solid structures or particles were present across all analyzed hair types. As a confirmation of achieving maximum extraction yield after overnight digestion, we also tested the digestion step up to 40 h. As the results obtained after 16 h and 40 h of digestions were in accordance, we concluded that the plateau was achieved before 16 h.

While it is not common for cortisol analysis in plasma, urine, saliva or mothers milk to use 2D LC–MS/MS, the hair matrix is vastly different and poses additional challenges for proper chromatographic separation of cortisol from interferences. With 2D LC–MS/MS compared to one-dimensional-LC–MS/MS, the chromatography improved significantly, while less clean-up steps were necessary, thus reducing the hands-on time even more. A twofold increase in the response was observed when switching from 1 to 2D LC–MS/MS, with an even higher increase for the signal to noise ratio since chromatography was much cleaner using two-dimensional chromatography. With the cleaner chromatography, automated integration was more consistent and less manual corrections were necessary than using the one-dimensional chromatography method, leading to more objective and accurate results.

The method was subsequently validated following the guidelines prescribed in the validation part of the “Methods” section. The precision was good in the homogenized pool, indicating that a larger, finely cut, and mixed hair pool can effectively serve as a quality control sample. This approach differs from strategies reported in the literature, where a larger hair quantity is extracted, the extract stored in aliquots, and then used as quality control; our strategy includes every aspect of sample handling, such as weighing small hair amounts. Moreover, our validation experiments showed sample stability after washing the hair and during storage at room temperature in the dark. As expected, duplicate analysis of intact hair locks yielded more variation than the homogenized control pool. The posterior vertex, the sampling region, has the lowest coefficient of intra-individual variation for hair cortisol concentration (indicating the least variation when cutting several locks from this region), but a mean CV of 15.6% (*n* = 28) is still reported [8]. Aliquoted hair locks (intact hair strands divided into smaller portions) are impacted by this inhomogeneity, while cortisol concentration in the homogenized control pool (finely cut and mixed hair samples used for quality control) is more consistent. This increased CV in the intact locks is not just due to analytical variation, but also results from preparing separate locks. Nonetheless, the precision results are still satisfactory and demonstrate that the protocol is suitable and replicable for cortisol analysis in various hair textures. The spike experiment, meant to assess accuracy, showed a greater variation than initially anticipated, particularly when spiking only a minor cortisol amount. We suspect this is partly due to the inhomogeneity following the aliquoting of the hair locks. However, with spike levels closer to the concentrations observed in hair samples, the recoveries were acceptable.

The sample preparation recovery (i.e., losses of cortisol and the internal standard ^13^C_3_-cortisol during preparation), matrix effect, and ion suppression were optimized and tested with this new protocol and were found to be acceptable. So far, no overlapping or closely eluting interferences have been observed in any of the prepared hair samples.

The analysis result using our new method is not affected by the quantity of starting material. Sixteen aliquots from the control pool were prepared in one experiment, encompassing a broad range of starting materials (5.8–38.6 mg of hair). This was evident from the linear plot (expected result based on the amount of prepared hair vs. measured result), which was obtained with an *R*^2^ value of 0.997.

The true recovery of cortisol from hair, i.e., the extraction yield, cannot be accurately assessed. However, we assume a complete or maximized recovery when the hair is fully dissolved. Our results on the recovery of spiked samples (taking into account endogenously present cortisol), and linearity of the measurements with a wide range of starting material, support our hypothesis that the extraction yield is very consistent across different hair types and amount taken into preparation, and increased compared to the current gold standard methanol extraction. Assuming a total extraction yield after digestion, we observed an average extraction yield of 81% using the methanol extraction protocol. This outcome supports our hypothesis that the extraction is incomplete with this extraction method, potentially leading to biased results.

Prepared samples can be stored before analysis. When comparing raw data (area cortisol/area internal standard) across different days, the CVs of the raw data were found to be lower than those obtained from the corrected results. This discrepancy is most likely because a fresh calibration curve was prepared and used for each experiment’s result correction. This evidences that if an experiment must be postponed, it is critical to store and re-analyze the same calibration curve from the original experiment, as generating new calibration curves introduces variations.

Hair cortisol has been identified as a biomarker for chronic stress, to provide a long-term retrospective overview of an individual’s past cortisol levels, not influenced by personal perception or recall bias. Cortisol levels are increased upon stress, and during chronic stress, the levels remain elevated for an extended period. There are several limitations to using hair cortisol as a biomarker for chronic stress, which do not stem from the analytical method, but rather from various factors affecting cortisol levels. Confounding factors previously identified include the frequency of hair washing, sampling season, age, gender, body mass index, and physical activity [7, 11, 17, 27–29]. Additionally, hair growth rates are not identical across individuals and may vary among ethnicities [28, 30] and different anatomical sites within the same individual [16]. This results in observed differences in cortisol concentration in hair from the chest, arms, and legs compared to scalp hair [31]. Some individuals, mainly men, cannot be sampled due to a lack of hair growth, which may introduce bias. These considerations should be kept in mind when interpreting any hair cortisol data.

The use of this biomarker has been validated through many clinical stress studies involving a comparison of groups. However, due to the previously mentioned limitations, employing this method as a biomarker to evaluate individual stress levels might be challenging if only slight increases or decreases in cortisol levels are noted, without prior knowledge of the individual’s baseline hair cortisol. This challenges the use of this method as a biomarker for individual stress level.

However, in a clinical context, this method can provide personalized information. It has the potential to confirm cyclic Cushing’s syndrome, characterized by repeated periods of hypercortisolism. By analyzing hair segments that correspond to different time periods, a rise in one or more specific segments could indicate temporarily elevated cortisol levels, as hair analysis allows for retrospective examination of cortisol levels over an extended period.

## Conclusion

We have developed and validated a new approach for the analysis of hair cortisol, focusing on a strategy meant to maximize the extraction yield of cortisol from the hair and to subsequently analyze the cortisol with great specificity. This approach provided a higher cortisol yield than methods utilizing methanol extraction, which has been predominantly used until now. The analytical method for determining hair cortisol concentration can serve as a biomarker for chronic stress in clinical studies.

The method may also be used in all conditions where cortisol levels are affected. As the enzymatic step does not require harsh or extreme conditions, it may be suitable for use in the analysis of other endogenous or exogenous compounds incorporated in hair as well.

## Supplementary Information

Below is the link to the electronic supplementary material.

ESM 1 (DOCX 31.1 KB)

## Data Availability

Data are available from the authors by request.

## Declarations

### Declarations

Hair samples were acquired, following informed consent, from adult female volunteers and drop-outs of a clinical study. In this study, healthy lactating females from the Netherlands, all aged over 18 years, were included. The study was conducted in accordance with the ethical standards and, where required, approved by the ethics committee of the Amsterdam UMC.
