# Supplementary material for: Enzymatic digestion of hair increases extraction yields of cortisol: a novel two-dimensional liquid chromatography-tandem mass spectrometry method for hair cortisol analysis
Source: Anal Bioanal Chem. 2025 Apr 16;417(15):3465–76. doi: 10.1007/s00216-025-05878-0 (PMC12122607; doi:10.1007/s00216-025-05878-0)
Supplement: Supplementary file 1 — (DOCX 31.1 KB) [file 216_2025_5878_MOESM1_ESM.docx]

Supplementary material

Enzymatic digestion of hair increases extraction yields of cortisol: A novel two-dimensional liquid chromatography-tandem mass spectrometry method for hair cortisol analysis

Analytical and Bioanalytical Chemistry

Dewi van Harskamp^1,2,3^, Mariëtte T. Ackermans^4^, Wjera V. Wickenhagen^3,5^, Annemieke C. Heijboer^3,5,6^, Johannes B. van Goudoever^6,7^

1. Amsterdam UMC, Department of Laboratory Medicine, Core Facility Metabolomics, Laboratory Genetic Metabolic Disease, University of Amsterdam, Amsterdam, Netherlands.
2. Amsterdam UMC, Emma Center for Personalized Medicine, Amsterdam, Netherlands.
3. Amsterdam UMC, Amsterdam Gastroenterology, Endocrinology & Metabolism, Amsterdam, Netherlands.
4. Ouderkerk aan de Amstel, the Netherlands
5. Amsterdam UMC, Department of Laboratory Medicine, Endocrine Laboratory, Vrije Universiteit Amsterdam, University of Amsterdam, Amsterdam, Netherlands.
6. Amsterdam UMC, Amsterdam Reproduction & Development Research Institute, Amsterdam, Netherlands.
7. Amsterdam UMC, Department of Pediatrics, Emma Children's Hospital, Vrije Universiteit Amsterdam, University of Amsterdam, Amsterdam, the Netherlands.

Corresponding author: Dewi van Harskamp, d.vanharskamp@amsterdamumc.nl

Equations for recovery and matrix effects

$\boldsymbol{Recovery} \boldsymbol{sample} \boldsymbol{preparation} \left( \% \right) = \frac{\boldsymbol{peak} \boldsymbol{area} IS \boldsymbol{added} \boldsymbol{before} \boldsymbol{sample} \boldsymbol{preparation}}{\boldsymbol{peak} \boldsymbol{area} IS \boldsymbol{added} \boldsymbol{after} \boldsymbol{sample} \boldsymbol{preparation}}\times\mathbf{100}\%$ Eq. (1)

$\boldsymbol{Matrix} \boldsymbol{effect} \left( \% \right) = \frac{\boldsymbol{peak} \boldsymbol{area} IS \boldsymbol{added} \boldsymbol{after} \boldsymbol{sample} \boldsymbol{preparation}}{\boldsymbol{peak} \boldsymbol{area} \boldsymbol{IS} \boldsymbol{of} \boldsymbol{spiked} \boldsymbol{solvent}} \times\mathbf{100}\%$ Eq. (2)

$\boldsymbol{Combined} \boldsymbol{matrix} \boldsymbol{effect} \boldsymbol{and} \boldsymbol{recovery} = \frac{\boldsymbol{peak} \boldsymbol{area} IS \boldsymbol{added} \boldsymbol{before} \boldsymbol{sample} \boldsymbol{preparation}}{\boldsymbol{peak} \boldsymbol{area} \boldsymbol{IS} \boldsymbol{of} \boldsymbol{spiked} \boldsymbol{solvent}} \times\mathbf{100}\%$ Eq. (3)

$$\boldsymbol{Spike} \boldsymbol{level} \left( \boldsymbol{observed} \right)=\boldsymbol{observed} \boldsymbol{result} \left[ \boldsymbol{pg} \right]-(\boldsymbol{sample} \boldsymbol{weight} \boldsymbol{of} \boldsymbol{aliquot} \boldsymbol{hair} \left[ \boldsymbol{mg} \right] \times\boldsymbol{concentration} \boldsymbol{of} \boldsymbol{cortisol} \boldsymbol{observed} \boldsymbol{in} \boldsymbol{unspiked} \boldsymbol{aliquot} [\boldsymbol{pg}/\boldsymbol{mg} \boldsymbol{hair}])$$

 Eq. (4)

Instrument settings

Columns:

First column: Acquity UPLC Protein BEH C4 (1.7 µm, 2.1 x 50 mm, Waters)

Second column: Acquity UPLC HSS T3 (1.8 µm, 2.1 x 50 mm, Waters)

Column heater: 50°C (first column) and 40°C (second column)

Mobile phase A: 2 mM ammonium acetate and 0.1% formic acid in water

Mobile phase B: 2 mM ammonium acetate and 0.1% formic acid in methanol

Wash solvent: 20% methanol in water

Temperature sample manager: 12°C

Gradient Quarternary Solvent Manager (gradient used in first column):

| time (min) | flow (mL/min) | Fraction A% | Fraction B% | Curve |
| --- | --- | --- | --- | --- |
| initial | 0.400 | 98 | 2 | Initial |
| 00:10 | 0.400 | 75 | 25 | 11 (step) |
| 04:30 | 0.400 | 2 | 98 | 11 |
| 05:80 | 0.400 | 2 | 98 | 11 |
| 05:90 | 0.600 | 98 | 2 | 11 |
| 07:90 | 0.600 | 98 | 2 | 11 |
| 08:00 | 0.200 | 98 | 2 | 11 |

Gradient Binary Solvent Manager (gradient used in second column):

| time (min) | flow (mL/min) | Fraction A% | Fraction B% | Curve |
| --- | --- | --- | --- | --- |
| initial | 0.400 | 2 | 98 | Initial |
| 02:00 | 0.400 | 2 | 98 | 6 (linear curve) |
| 02:10 | 0.400 | 75 | 25 | 6 |
| 04:90 | 0.400 | 75 | 25 | 6 |
| 04:95 | 0.400 | 54 | 46 | 6 |
| 07:95 | 0.400 | 48 | 52 | 6 |
| 08:00 | 0.200 | 2 | 98 | 6 |

MS/MS parameters

| Source | ion mode | ESI+ |
| --- | --- | --- |
|  | capillary voltage | 0.6 kV |
|  | cone voltage | 40 V |
|  | source offset | 60 V |
|  | source temperature | 150°C |
|  | desolvation temperature | 625°C |
|  | desolvation gas flow (N2) | 1000 L/uur |
|  | cone gas flow (N2) | 150 L/uur |

MRM settings

| component | m/z | dwell time (s) | cone (V) | col energy | |
| --- | --- | --- | --- | --- | --- |
| cortisol Quantifier | 363 -> 121 | 0.088 | 40 | 21 | |
| cortisol qualifier | 363 -> 309 | 0.088 | 40 | 15 | |
| cortisol qualifier 2 | 363 -> 327 | 0.088 | 40 | 13 | |
| cortisol ^13^C_3_ (IS) | 366 -> 124 | 0.088 | 40 | 21 | |
| Cortisol ^13^C_3_ qualifier | 366 -> 272 | 0.088 | 40 | 18 | |
| Retention window (min) | | 6 min to 9.5 min | | | |
